# Low Maternal Capital Predicts Life History Trade-Offs in Daughters: Why Adverse Outcomes Cluster in Individuals

**DOI:** 10.3389/fpubh.2019.00206

**Published:** 2019-07-31

**Authors:** Jonathan C. K. Wells, Tim J. Cole, Mario Cortina-Borja, Rebecca Sear, David A. Leon, Akanksha A. Marphatia, Joseph Murray, Fernando C. Wehrmeister, Paula D. Oliveira, Helen Gonçalves, Isabel O. Oliveira, Ana Maria B. Menezes

**Affiliations:** ^1^Policy, Population and Practice Programme, UCL Great Ormond Street Institute of Child Health, London, United Kingdom; ^2^Faculty of Epidemiology and Population Health, London School of Hygiene and Tropical Medicine, London, United Kingdom; ^3^Department of Community Medicine, UiT the Arctic University of Norway, Tromsø, Norway; ^4^Department of Geography, University of Cambridge, Cambridge, United Kingdom; ^5^Federal University of Pelotas – Postgraduate Program in Epidemiology, Rua Marechal Deodoro, Pelotas, Brazil

**Keywords:** maternal investment, life history theory, trade-offs, reproduction, growth, education, inter-generational effect, obesity

## Abstract

**Background:** Some individuals appear prone to multiple adverse outcomes, including poor health, school dropout, risky behavior and early reproduction. This clustering remains poorly understood. Drawing on evolutionary life history theory, we hypothesized that maternal investment in early life would predict the developmental trajectory and adult phenotype of female offspring. Specifically, we predicted that daughters receiving low investment would prioritize the life history functions of “reproduction” and “defense” over “growth” and “maintenance,” increasing the risk of several adverse outcomes.

**Methods:** We investigated 2,091 mother-daughter dyads from a birth cohort in Pelotas, Brazil. We combined data on maternal height, body mass index, income, and education into a composite index of “maternal capital.” Daughter outcomes included reproductive status at 18 years, growth, adult anthropometry, body composition, cardio-metabolic risk, educational attainment, work status, and risky behavior. We tested whether daughters' early reproduction (<18 years) and exposure to low maternal capital were associated with adverse outcomes, and whether this accounted for the clustering of adverse outcomes within individuals.

**Results:** Daughters reproducing early were shorter, more centrally adipose, had less education and demonstrated more risky behavior compared to those not reproducing. Low maternal capital was associated with greater likelihood of the daughter reproducing early, smoking and having committed violent crime. High maternal capital was positively associated with the daughter's birth weight and adult size, and the likelihood of being in school. Associations of maternal capital with cardio-metabolic risk were inconsistent. Daughters reproducing early comprised 14.8% of the population, but accounted for 18% of obesity; 20% of violent crime, low birth weight and short stature; 32% of current smoking; and 52% of school dropout. Exposure to low maternal capital contributed similarly to the clustering of adverse outcomes among daughters. Outcomes were worst among daughters characterized by both low maternal capital and early reproduction.

**Conclusion:** Consistent with life history theory, daughters exposed to low maternal capital demonstrate “future discounting” in behavior and physiology, prioritizing early reproduction over growth, education, and health. Trade-offs associated with low maternal capital and early reproduction contribute to clustering of adverse outcomes. Our approach provides new insight into inter-generational cycles of disadvantage.

## Introduction

There is major public-policy interest in investing in children, in order to allow them to achieve their full potential in health and human capital, and flourish in adult life. This is particularly relevant to high-risk groups, who have been shown to contribute disproportionately to adverse adult outcomes. For example, a recent analysis of the Dunedin cohort of ~940 individuals showed that “a segment comprising [only] 22% of the cohort accounted for 36% of the cohort's injury insurance claims; 40% of excess obese kilograms; 54% of cigarettes smoked; 57% of hospital nights; 66% of welfare benefits; 77% of fatherless child-rearing; 78% of prescription fills; and 81% of criminal convictions” (1). As childhood risks predicted these adult outcomes, the authors concluded that early interventions targeting this high-risk segment of the population could yield large returns on investment.

This study has important implications for public health. It highlights the benefits of considering a wide range of outcomes, embracing both health and human capital outcomes, within a single conceptual framework, and in addition, indicates that the clustering of adverse traits in adulthood has a developmental origin. Nevertheless, the study also raises two related questions: first, we still need to explain *why* diverse adverse outcomes encompassing both physiological and behavioral traits should cluster within individuals; second, it remains unclear *how early* in the life-course interventions should be targeted, in order to maximize their benefits. We develop an evolutionary approach to address the first question, which has additional implications for the second.

Physiological and behavioral outcomes are often addressed using very different conceptual approaches, reflecting contrasting theoretical perspectives widely used by biomedical or social scientists. However, a unique integrative framework is provided by evolutionary life history theory, which aims to predict phenotypic variability in general (2, 3), and which can therefore address both physical and behavioral traits. This theory assumes that organisms are under selective pressure to harvest resources from the environment throughout the life-cycle, and to allocate them to biological functions to maximize fitness (4). Energy is allocated between competing functions (maintenance, growth, reproduction and defense), resulting in trade-offs between them (5).

Most often, life history theory is used by biologists, in order to explain inter-species variability in traits such as reproductive strategy and longevity. For example, it has long been assumed that species exposed to high extrinsic mortality risk are unlikely to reap substantial pay-offs from investing in growth and maintenance, and will instead maximize fitness by prioritizing immediate survival and reproduction (6). Such organisms therefore mature rapidly, and produce numerous offspring, few of which survive to adulthood. As extrinsic mortality risk falls, the pay-offs from investing in maintenance increase, thus extending average longevity (7). Although this model is broadly supported, recent work suggests that the association between mortality risk and lifespan is more complex, and may depend for example on when in the lifespan mortality risk is greatest (8). Nevertheless, this framework can explain the clustering of growth-, reproduction-, and longevity-related traits across species, with the trade-offs orchestrated through genetic adaptation (6).

However, life history theory may also be applied to investigate variability within a species, and for example has been invoked by biologists to explain the clustering of behavioral traits, offering an adaptive perspective on “animal personalities” (9). This approach assumes that individuals vary in the relative importance allocated to current vs. future reproductive opportunities, resulting in populations with contrasting strategies for risk-taking behavior. Individuals prioritizing future reproduction are expected to be risk-averse in multiple different contexts, in order to realize those future opportunities, whereas those prioritizing immediate reproduction are predicted to discount the long-term future and to be more risk-prone in a range of contexts.

While genetic factors contribute to variability in life history traits, plasticity is also relevant. Through norms of reaction (whereby a single genotype can give rise to a range of possible phenotypes, depending on the ecological stimuli or stresses encountered) (10) physiological and behavioral trade-offs may emerge within the life-course under the common logic of future discounting, and this approach can be extended to humans. Regarding human behavior, for example, individuals exposed to harsh or unpredictable environments may have less incentive to invest in their health or education if they feel unlikely to realize the benefits at a later date (11, 12). Regarding physiology, the “disposable soma” theory likewise assumes that the lower the likelihood of survival, the lower the pay-off from investing in cellular health (7, 13). Constraints on somatic development may therefore undermine long-term health by favoring the prioritization of immediate survival and reproduction, increasing the rate of damage-accumulation with age (14, 15).

There is increasing interest in applying life history theory to both physiology and behavior in humans (16). Nonetheless, attempts to address phenotypic plasticity in this context remain scarce, and the results to date have been mixed (17). The exposures most commonly studied relate to post-natal life, and include behavioral markers of parental investment or extrinsic mortality risk (18, 19). In most studies, the outcome is also a single life history function, such as reproductive schedule. We suggest that these approaches neglect other important environmental factors that induce differential responses during early development, including those acting during prenatal life, and that previous studies have rarely considered competition across life history functions.

We have developed a conceptual approach emphasizing that the initial source of environmental variability experienced by placental mammals derives not directly from the external environment, but from maternal phenotype (20). During the earliest stages of development, often termed “critical windows of development,” maternal phenotype represents a “safe harbor” (21) that can buffer the vulnerable fetus and infant from external stresses through multiple components of homeostasis (20). Building on the “embodied capital” model (22), we refer to “maternal capital” as a suite of traits promoting investment in offspring in early life (20). Maternal capital incorporates not only somatic traits (e.g., height, pelvic dimensions, energy stored in adipose tissue), physiological traits (e.g., homeostatic regulatory processes), social traits (e.g., supportive networks of kin or peers), cognitive traits (e.g., knowledge and skills acquired from formal education, or informally), and psychological traits (e.g., resilience to psychosocial stress), but also material assets (e.g., income, savings, housing etc.) that may benefit the offspring. Maternal capital therefore represents a broad composite 'niche' to which the offspring is exposed during early life (20). Since phenotypic plasticity is greatest in these early life periods, exposure to varying levels of maternal capital may generate long-lasting effects on offspring phenotype.

The magnitude of maternal capital may vary markedly within a population, through the differential exposure of mothers to factors such as poverty, malnutrition, infectious disease and gender inequality (20, 23), as well as life history trade-offs that emerge during maternal development (24). Any insult to the “safe harbor” may therefore propagate effects into the next generation. Our over-arching hypothesis is that maternal investment, a function of the magnitude of maternal embodied capital, may leave a unique imprint on the offspring during their early stages of development, driving subsequent trade-offs in both behavioral and physiological traits. In turn, the emergence of multiple trade-offs might result in the clustering of beneficial traits within some individuals, and the clustering of adverse traits among others.

**Figure 1:**
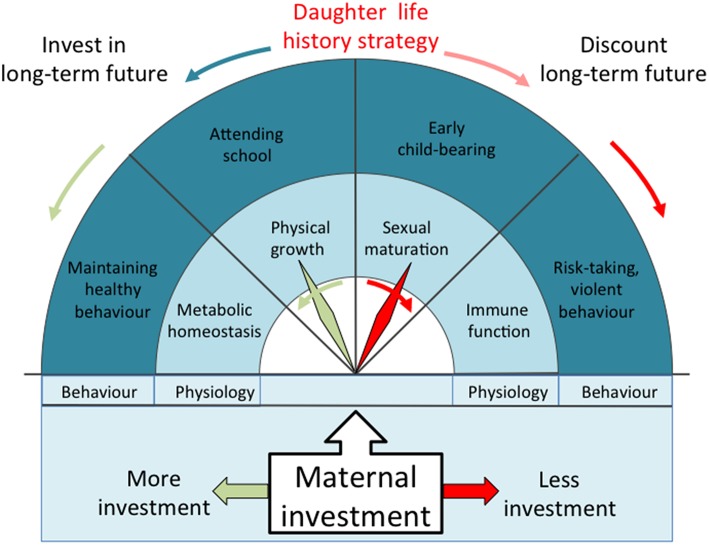
Schematic diagram illustrating our theoretical model derived from life history theory. The greater the magnitude of maternal investment during early life, the higher the phenotypic quality of the offspring and the greater the expected returns in the long-term future. Offspring fitness would then be maximized by investing in growth, maintaining health, acquiring education, and avoiding risky behavior, delaying reproduction until these other benefits have been realized. Lower maternal investment reduces the likely long-term returns, and favors earlier reproduction at a cost to competing traits. Central abdominal fat indicates greater allocation of energy to immune function, but at a cost to cardio-metabolic health.

Such trade-offs may be especially relevant in hierarchical populations, in which social inequality is propagated over generations (23, 25). Subordinate individuals are likely to experience reduced access to crucial resources throughout the life-course, generating the prediction that they must reorganize their life history strategy in order to maximize reproductive fitness under these harsh conditions (23). Specifically, we hypothesize that lower maternal investment will induce trade-offs in the offspring that favor early reproduction to counter the elevated mortality risk (7), elevated immune defense to improve resistance to infectious disease (26), and more risk-prone behavior due to discounting of any long-term consequences (11, 12), at a cost to each of somatic growth, markers of cardio-metabolic health associated with homeostasis, and investment in education (Figure 1). We lacked direct markers of immune function, and used central fat distribution as a marker of storing energy for this function (for example, many genes associated with immune function are highly expressed in visceral fat, and low levels of leptin, a hormone secreted by adipose tissue, predicts mortality following severe malnutrition) (27–29). Through these cumulative trade-offs, we predict the clustering of adverse outcomes among those exposed to low maternal investment.

We tested these hypotheses in a large prospective birth cohort in southern Brazil, a country with very high levels of social inequality. We used early child-bearing (<18 years) as a key marker of life history trade-offs among the daughters, as reproduction before 18 years is associated with health risks and other penalties among both mothers and offspring (30), and is widely discouraged by policy-makers (31), while reproduction is the currency of fitness from an evolutionary perspective. We restricted our analyses to mothers and daughters, reflecting our interest in “maternal capital” as the key exposure, and because trade-offs in male offspring might show different patterns.

## Methods

The birth cohort we studied is located in the city of Pelotas (~334,000 inhabitants), in the southern Brazilian state of Rio Grande do Sul. According to data for 1991, shortly before the cohort was established, 91.6% of Pelotas inhabitants lived in urban areas, the crude birth rate was 19.3 births per 1,000 population, the Human Development Index was 0.558 and the Gini index for income distribution was 0.59 (32)^1^.

### Cohort Profile

The birth cohort was established in 1993, when all mothers who delivered a newborn in the five hospitals of the city and who resided in the urban area were invited to participate in a birth cohort study (99% of all births in the city area) (33). Through daily visits to all 5 hospitals, data were collected on 5,249 live births (both sexes) and only 16 individuals (0.3%) refused to participate. The cohort participants have been followed up at different time points thereafter. All visits were carried out by trained interviewers and fieldwork team members. Further details of the methodology have been published previously (33, 34). Among those not followed, small numbers had died or refused to participate, however the majority of losses were due to individuals not being traced, or having moved to other cities.

In all phases of the study, ethical approval was obtained from the Medical School Ethics Committee of the Federal University of Pelotas and full informed consent was provided by parents or their legal representatives (if the subject was aged under 18 years) or by cohort members. Verbal consent was provided in the perinatal phase.

### Data Collection

At the initiation of the study when the cohort members were born, data were collected on maternal phenotype including nutritional status, behavioral traits, reproductive history, educational attainment and family circumstances (33). A strength of the study is its prospective nature, such that maternal phenotype was measured at the start of the life-course of the offspring.

Data on family characteristics, pregnancy exposures, and early breast-feeding were obtained by questionnaires administered while the mother was still in hospital, and in a subsample at a 1 year follow-up. Family income at the time of birth was assessed in units of minimum wages (in 1993, 1 minimum wage = US$ 31.4 per month). Maternal smoking and alcohol consumption during pregnancy were collected retrospectively at birth. Gestational age was recorded in weeks. Exclusive breast-feeding was recorded in days. Maternal parity was recorded, and was used to create several dummy variables for analysis as described in more detail below. Maternal age was recorded in relation to the date of the daughter's birth.

Offspring birth weight and length were measured at the hospital by the research team. Weight and length at 12 months were measured in a subsample at the cohort participant's household. Information on the daughter's age at menarche was obtained from the mother at the 15 years visit, and confirmed by the subject at the 18 years visit. Age at menarche was assessed as the age of occurrence of the first menstrual cycle.

At 18 years, weight and height were measured and used to calculate body mass index (BMI). Waist circumference was measured using a non-elastic measuring tape. Fat-free and fat mass were assessed using air-displacement plethysmography.

Cardiovascular risk markers measured at the 18-year follow-up included: glucose, glycated hemoglobin (HbA1c), total cholesterol (TC), HDL-cholesterol (HDL-C), LDL-cholesterol (LDL-C), triglycerides (TGL), and systolic (SBP) and diastolic (DBP) blood pressure. The ratio of total cholesterol to HDL was also calculated, with higher values indicating a less favorable cholesterol profile. Venous blood samples were collected regardless of fasting status, left at room temperature for 30 min and then centrifuged for 15 min at 2,000 g. Serum aliquots were stored at −80°C until analysis. Blood samples were not taken in pregnant or suspected pregnant participants (*n* = 59). Random glucose was measured by an automatic enzymatic colorimetric method. HbA1c was measured by the Variant (Bio-Rad, Hercules, CA) ion-exchange high-performance liquid chromatography (HPLC) method. Lipids were measured using an automatic enzymatic colorimetric method in a biochemistry analyzer (BS-380 Mindray; Shenzhen Mindray Bio-Medical Electronics, China). We excluded two individuals with implausibly high triglyceride values. Blood pressure was recorded in the seated position using a calibrated digital wrist monitor (Omron HEM-629, Beijing, China) at the start and end of the visit, and the mean of the measurements used in the analysis.

A questionnaire was used to ascertain reproductive status at 18 years. For those who had reproduced, information was collected on the number of live offspring, the age at delivery, and the birth weight of the infant.

A questionnaire was used to establish schooling status at 18 years, including categorical data (whether studying now; whether studied in the last year) and continuous data (completed years of education). For those not studying (*n* = 317), participants were asked to select from a list of 10 possible reasons accounting for this: difficulty learning; illness; work; no school or travel available; education not considered important; having children; married; violence; failed vestibular examination; other.

Participants were asked if they received any income from work, or an allowance (usually from parents) and the amount in Reais (Rs). Questionnaires were also used to establish smoking behavior, and whether the participant had ever committed a violent crime.

### Data Processing

We categorized daughters according to whether or not they had reproduced early (by 18 years) or not.

We categorized mothers according to the magnitude of their capital. This approach combined markers of somatic capital (height, pre-pregnancy BMI) and social or material capital (maternal education and family income) into a composite index. Each of these four traits has been widely associated with phenotypic variability in the next generation (35–38). For each trait, a cut-off was identified defining approximately the lower tertile in the population, in order to identify those substantially below the median, as follows:

Height: <157 cm
Pre-pregnancy BMI: <21 kg/m^2^
Maternal education: <6 years
Family income: <3 minimum salaries

The four dummy variables were then summed, allowing mothers to be assigned a score based on the number of “capital penalties” with values ranging from 0 to 4 (i.e., mothers with high capital had few capital penalties). We used this variable to explore continuous associations of maternal capital with daughter outcomes. In addition, those with a score of 4 were categorized as the “low” composite maternal capital group, while those with a score of 0 categorized as the “high” composite maternal capital group. This allowed us to compare outcomes of daughters between these two contrasting maternal capital groups. For logistic regression analyses, we also divided maternal age into three groups, namely <22, 22–28, and >28 years. For the same purpose, we divided the daughters into three groups in relation to maternal parity, namely first-borns, second-borns, and third^+^-borns. However, for descriptive analysis we also generated a category for high maternal parity, defined as the daughter being fourth/fifth-born.

To assess clustering of adverse traits among the daughters, we defined several categorical variables. At 18 years, we defined obesity as BMI >30 kg/m^2^, short stature as height <157 cm, and school dropout as those reporting not studying during the last year. Self-reported current smoking and having committed violent crime were additional adverse outcomes. Finally, we included low birth weight as an adverse outcome, as those with this characteristic remain at elevated risk of non-communicable disease through adult life (39). We defined “low birth weight” as <2,500 g.

### Analytical Steps

**Figure 2:**
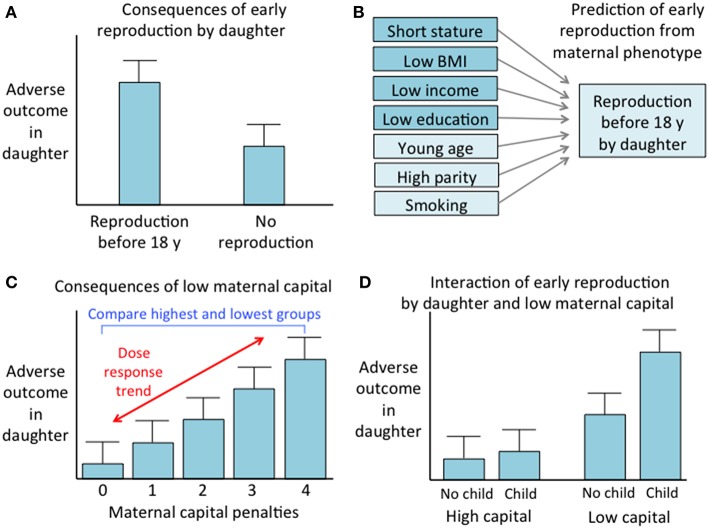
Conceptual diagram of the analytical steps. **(A)** Comparison of outcomes between daughters who reproduced before 18 years vs. those who did not. **(B)** Prediction of early reproduction by daughter from components of maternal capital, including those used to generate the maternal capital index and three other maternal traits. **(C)** Analysis of the association of adverse daughter outcomes with maternal capital, involving both a comparison of mothers with 0 or 4 maternal capital penalties, and assessment of a dose-response trend across the full range of maternal capital penalties. **(D)** Analysis of the interactive associations of early daughter reproduction and low maternal capital with adverse daughter outcomes.

We undertook several analytical steps, which are summarized in a conceptual diagram (Figure 2).

- Our first analytical step was to test for inverse associations in the daughters between early reproduction (<18 years) and markers of growth, health and education, which we interpret as trade-offs as predicted by life history theory. For this purpose, we analyzed several broad groups of outcomes of the daughters. The first comprised growth trajectory, comprising birth weight, infant growth, age at menarche, and adult size (height, fat-free mass, fat mass and its regional distribution). The second comprised markers of metabolic health, blood pressure and blood biochemistry. The third comprised behavioral profile, focusing on education, income, and risky behavior (smoking). We used these outcomes to assess relative investment during development in the competing life history functions of growth, maintenance and reproduction. Markers of risky behavior (smoking, committing violent crime), were considered indicative of future discounting. We conducted these analyses by comparing daughters with vs. without offspring at 18 years, using independent samples *t*-tests or chi-squared tests. To test for “developmental origins” of these trade-offs, we extended the comparison to the daughters' growth trajectories in early life. To begin to test our key prediction that maternal capital is associated with these trade-offs, we also extended the comparison to the characteristics of their mothers.
- Our second analytical step tested our novel “maternal capital” model, by defining groups of mothers with different composite levels of maternal capital and quantifying associations with the daughters' phenotype. Using the composite maternal capital index, we tested the hypothesis that variability in maternal phenotype predicted the likelihood of early reproduction by the daughter, and hence also predicted variability in the traits that either trade off against early reproduction, or correlate directly with it. We first fitted logistic regression models to confirm that multiple individual maternal traits predicted reproductive status of the daughter (had a child or not by age 18 years). These models included the four variables used to generate the composite index of maternal capital, but also additional markers of the capacity for maternal investment (age and parity) as well as “risky maternal behaviors” (smoking during pregnancy) that might constrain maternal investment.
- Third, we then ran regression models testing dose-response associations of daughter traits with the number of maternal capital penalties. We highlighted the contrast between daughters of low- vs. high-capital mothers using graphic analysis, supported by independent-sample *t*-tests. Outcomes included the daughters' gestational age and early growth trajectory, breast-feeding experience, adult size and body composition, cardio-metabolic risk markers, risky behaviors, educational attainment, and early onset of reproduction, as well as maternal parity and the mother's risky behavior (smoking, alcohol intake). For growth, we were able to assess whether differences extended to the third generation, by analyzing birth weight of the daughter's own offspring.
- Our fourth analytical step followed the approach of Caspi et al. (1) to evaluate the extent to which adverse outcomes were clustered among the minority of the daughters who had reproduced by 18 years, and among those who had been exposed to low maternal capital. We analyzed dichotomous outcomes for obesity (>30 kg/m^2^), short stature (<157 cm), current smoking, school dropout, violent crime, and low birth weight, as justified above. We also explored the interactive associations of low maternal capital and early reproduction by the daughter with these adverse daughter outcomes.
- Associations between early reproduction in daughters and adverse outcomes might potentially be confounded by direct mother-daughter transmission of the outcome, due to shared genes or household environments. To exclude this possibility, we fitted regression models in which associations of the daughter's early childbearing with various outcomes were adjusted for equivalent traits in the mother.

### Statistical Analysis

Of the 2,645 female participants, 2,091 participated in the follow-up and provided questionnaire responses, of whom 2004 also underwent the physical measurements of anthropometry and body composition, and 1936 completed the blood test. There were small numbers of missing data for individual variables, hence we used all available data for every analysis. The subsample at 1 year (*n* = 723) over-sampled those with low birth weight. This was addressed using a weighting variable in the relevant regression models.

Categorical outcomes were assessed using chi-squared tests and odds ratios. Conditional growth was calculated as regression residuals of final size on starting size, divided by the standard error of the estimate (SEE) of the regression model to generate conditional *z*-scores. Continuous outcomes reported in the main figures were all natural-log transformed, so that subsequent independent samples *t*-tests express differences (multiplied by 100) in sympercent terms (40).

To establish which maternal factors independently predicted daughters' reproduction status at 18 years, logistic regression models were fitted. Maternal capital was divided into groups as follows, in order to identify high-risk groups and test for threshold effects:

Age <22, 22–28, 28+ years
Height <155, 155–162, 162+ cm
Education 0–4, 5–7, 8+ y
Income 0–2, 3–4, 5+ minimum wages
Parity First-born, second-born, third^+^-born
Smoking in pregnancy Yes, no
Maternal BMI <21, 21–23.5, 23.5+ kg/m^2^

Logistic regression models were also used to test the association of daughter phenotype with the odds of early reproduction, without or with adjustment for the equivalent trait in the mother.

To test the interactive associations of low maternal capital and early daughter reproduction with adverse daughter outcomes, we conducted chi-squared tests on dichotomous variables (e.g., being short, out of school etc.). We compared simple exposures (2 groups, either high vs. low maternal capital, or early reproduction vs. no early reproduction) against a composite exposure (4 groups, differentiating reproductive status among each maternal capital groups). Due to the small sample size of the lowest maternal capital group (4 capital penalties), we combined the 3 and 4 capital penalty groups to give a larger “low maternal capital” group for this analysis and compared them against those with 0 capital penalties. We used the likelihood ratio value to compare these models for their ability to explain variability in the dichotomous outcomes.

All analyses were conducted in SPSS version 24 (IBM Corporation, Chicago) and R version 3.4.1 (The R foundation for statistical computing, Vienna).

## Results

A total of 2,091 female cohort participants (the daughters) were followed up at 18 years, representing a 79.0% retention rate of the 2,645 individuals originally recruited at birth. Those not followed up had lower birth weight (Δ = −103 g, 95%CI −53, −152), birth length (Δ = −0.3 cm, 95%CI −0.1, −0.5), gestational age (Δ = −0.3 weeks, 95%CI −0.1, −0.4) and maternal pre-pregnancy BMI (Δ = −0.4 kg/m^2^, 95%CI −0.1, −0.8) compared to those followed up. Though statistically significant, these differences were all of small magnitude, and no other differences in baseline maternal or child characteristics were evident (Supplementary Table 1).

### Missing Data Analysis

At 18 years, the proportion of missing data for daughter outcomes was as follows: 0% for reproductive status, education, work status and smoking status; 0.1% for birth weight and age at menarche; 0.9% for birth length; 1.5% for gestational age; 4.2% for the physical examination (blood pressure, anthropometry, and body composition); 7.4% for blood biochemistry; and 11.7% for violent crime. These missing outcome data could not be imputed, hence *t*-tests were used to assess whether these data were missing at random in relation to maternal capital predictors.

For birth weight, birth length, age at menarche, and gestational age, data was missing at random. Daughters (*n* = 87) missing data from the physical examination at 18 years (anthropometry, body composition, and blood pressure) had mothers who were shorter (−1.7 cm, *p* = 0.018), younger (−1.4 years, *p* = 0.04), and less educated (−0.9 years, *p* = 0.015) compared to the mothers of daughters with complete data, but no other differences were apparent. Daughters (n = 155) missing data from the blood sampling did not demonstrate any differences in maternal phenotype compared to daughters with complete data. Daughters (*n* = 244) missing data on violent crime had mothers who had lower income (−0.6 minimum wages, *p* = 0.047) compared to the mothers of daughters with complete data, but no other differences were apparent.

The proportion of missing data for maternal predictors was low, being 0% for parity, smoking status and age; 0.1% for education; 0.5% for height; 2.0% for income; and 1.6% for weight. Collectively, this meant that 2.1% lacked data on BMI. Multiple imputation was implemented to address these missing predictor data. Five imputed datasets were generated, and the results are discussed below.

### Life-History Trade-Offs in Association With Early Reproduction of Daughters

**Figure 3:**
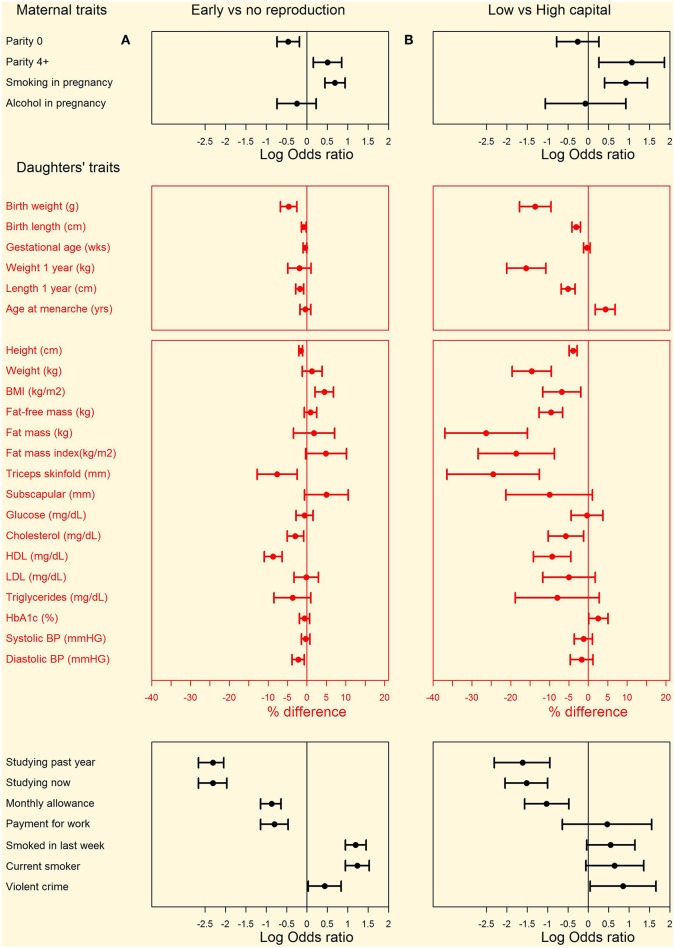
Differences in maternal and daughter traits from pregnancy to adulthood between **(A)** left hand panel: daughters with or without offspring by 18 years and **(B)** right hand panel: low-capital and high-capital mothers. Categorical variables are shown as odds ratios and 95% confidence intervals, calculated by chi-square tests. Continuous variables are shown as percent differences and 95% confidence intervals, calculated from natural log-transformed variables. Numerical values for all comparisons are given in Supplementary Table 2.

**Figure 4:**
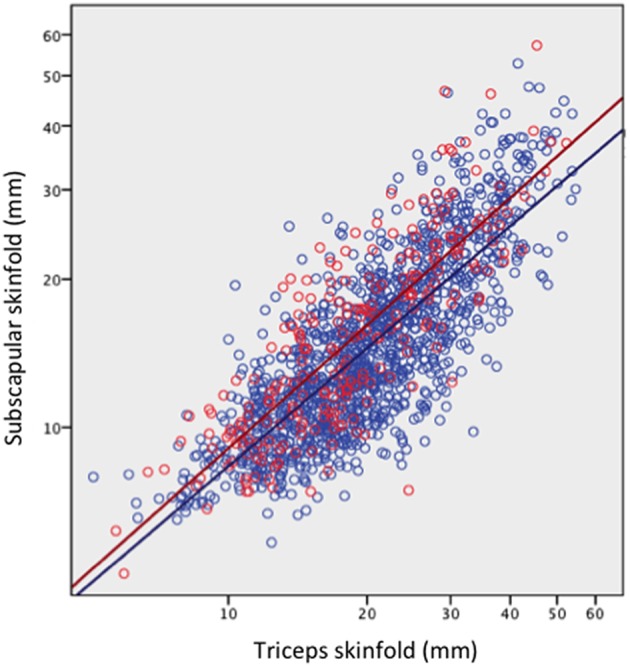
Subscapular skinfold plotted against triceps skinfold in the adult daughters, stratified by whether they had reproduced by 18 years (red scatter and line) or not (blue scatter and line). Early reproducing daughters have significantly higher subscapular skinfold (Δ = 2.0 mm, 95%CI 1.5, 2.6) for a given triceps skinfold, indicating a more central fat deposition.

The comparison of daughters who had or had not reproduced by 18 years is summarized in Figure 3A (see Supplementary Table 2 for numerical values). Early-reproducing daughters were shorter (Δ = 2.5 cm) than those without offspring, but similar in weight. They had higher BMI (Δ = 1.0 kg/m^2^) and were more likely to be obese (OR 1.5, 95%CI 1.1, 2.2). They had higher total fat mass index, however this was accompanied by lower triceps but higher subscapular skinfold, in combination indicating a more central fat deposition (Figure 4). They had lower total cholesterol and HDL, and a less favorable ratio of total cholesterol to HDL (Δ = −0.19, 95%CI −0.26, −0.12), but otherwise showed no differences in cardio-metabolic health.

**Figure 5:**
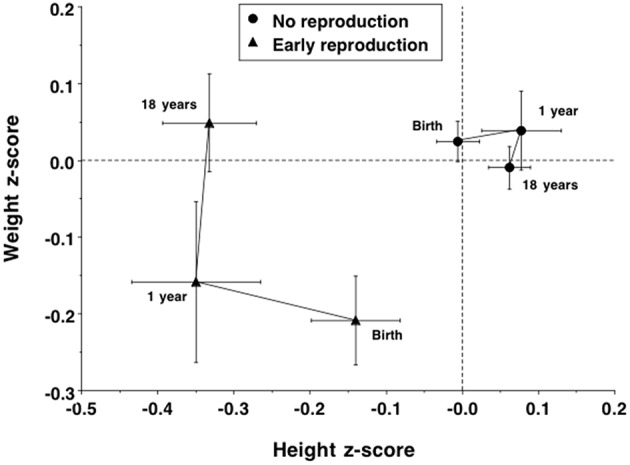
Trajectories of growth in length/height z-score and weight z-score in the daughters, stratified by whether they had reproduced by 18 years or not. Early reproducing daughters showed poor linear growth between birth and 1 year, but increased in weight between 1 and 18 years, resulting in their achieving similar weight at 18 years to non-reproducing daughters at a cost to growth in height.

These differences in adult size were associated with contrasting growth trajectories. Daughters with children had been, at birth, significantly shorter (Δ = 0.4 cm) and lighter (Δ = 143 g), and had shorter gestation length (Δ = 0.2 weeks). Daughters with children also grew significantly less in length between birth and 1 year (−0.34 conditional *z*-scores, 95%CI −0.63, −0.16), but similarly in weight, while in adolescence the two groups had similar age at menarche. Overall, the early-reproducing daughters showed a growth trajectory favoring weight gain at the expense of linear growth (Figure 5), and the contrast in height had already reached its adult magnitude in *z*-score terms by 1 year of age.

Daughters bearing children early were much less likely than those still childless to have studied over the last year, or to be in school at the time of the follow-up, and had completed on average 2.4 (95%C1 2.2, 2.7) years less education. Among a subset with relevant data (n = 317), the most common reason that daughters with children gave for not being in school was being a mother (69%) or being married (10%), whereas for those without children, the most common reasons were work (37%), or not considering education important (17%) (Table 1). When those giving the response “having children” were omitted from this analysis (since this response was only relevant to one group), the chi-square test remained significant (*p* < 0.0001) and the strongest contrasts were daughters without children being more likely to have left school to work, for marriage, or for an unstated reason.

**Table 1:** Reasons for not studying, stratified by daughters' early reproduction status.

	**Have 1+** **child (*****n*** **=** **157)**		**No children (*****n*** **=** **160)**		**P^b^**
**Reason for not studying^a^**	***N***	**%**	***N***	**%**	
Difficulty learning	3	1.9	7	4.4	<0.0001
Illness	1	0.6	7	4.4	
Work	7	4.5	60	37.5	
No school or travel available	1	0.6	10	6.3	
Education not considered important	10	6.4	27	16.9	
Having children	108	68.8	0	0	
Married	15	9.6	13	8.1	
Violence	0	0	2	1.3	
Failed vestibular examination	1	0.6	15	9.4	
Other	11	7.0	19	11.9	

a *Analysis of cohort subsample (n = 317) with detailed data on education status*.

b *p-value applies both to analyses including all adolescents with children, or excluding those who cited “having children” as this response was not relevant to the other group*.

Daughters with children were less than half as likely to have received a monthly allowance (in Brazilian Reais, Rs) or payment for work in the last month, though if they had, the amount was similar to that received by daughters without children (allowance: Δ = −13 Rs, 95%CI −28, 53; work payment: Δ = −29 Rs, 95%CI −47, 92). They were more than three times as likely to declare themselves current smokers, and to have smoked at least once during the last week, and 1.5 times more likely to have committed violent crime.

The two groups further showed significant contrasts in the characteristics of their mothers. Compared to those without children, early-reproducing daughters with children were less likely to be first-born and more likely to be fourth^+^-born. Their mothers were twice as likely to have smoked in pregnancy, but were no different in terms of alcohol intake. Their mothers were on average significantly younger (Δ = −1.6 y; 95%CI −0.9, −2.8), shorter (Δ = −2.0 cm; 95%CI −1.2, −2.8), poorer (Δ = −2.1 minimum wages; 95%CI −1.7, −2.5), and had less education (Δ = −2.1 y; 95%CI −1.8, −2.5), but they did not differ in BMI (−0.27 kg/m^2^; −95%CI −0.73, 0.18).

These findings were essentially unchanged if multiple imputation was used to address the small proportion of missing data relating to maternal predictors.

Overall, early reproduction by daughters was associated with poorer growth and educational attainment, a more unhealthy distribution of body fat, and an increased likelihood of risky behavior. These trade-offs appeared to have their origins in early life, indicated by associations of early reproduction with growth trajectory and with maternal capital indicators.

### Which Components of Maternal Capital Predict Daughter's Early Reproduction?

Table 2 reports the results of logistic regression models, testing for independent associations of maternal characteristics with the risk of early reproduction by the daughter. Young age, short height, low education, low income, higher parity, and smoking were all independent maternal predictors, whereas low maternal BMI was not.

**Table 2:** Multivariable logistic regression testing independent associations of maternal capital components with odds of daughter reproducing by 18 years^a^.

**Maternal capital component**	**NK *r*^**2**^ = 0.160**		
	**Exp (coeff)**	**95%CI**	***p*****-value**
**Age (28+** **years** **=** **reference)**			
<22 years	2.53	1.52, 4.20	<0.0001
22–28 years	1.78	1.22, 2.57	0.002
**Height (162+** **cm** **=** **reference)**			
<155 cm	1.79	1.19, 2.68	0.005
155–162 cm	1.49	1.03, 2.16	0.036
**Education (8+** **years** **=** **reference)**			
0–4 years	2.66	1.64, 4.30	<0.0001
5–7 years	2.06	1.29, 3.30	0.003
**Income (5+** **minimum wages** **=** **reference)**			
0–2 minimum wages	3.46	1.94, 6.18	<0.0001
3–4 minimum wages	3.58	1.95, 6.58	<0.0001
**Parity (first-born** **=** **reference)**			
Second-born	1.31	0.82, 2.08	0.19
Third-born or higher	2.07	1.31, 3.29	0.016
**Smoking in pregnancy (no** **=** **reference)**			
Yes	1.41	1.03, 1.93	0.032
**Maternal BMI (23.5+** **kg/m**^**2**^ **=** **reference)**			
<20 kg/m^2^	1.17	0.76, 1.80	0.19
20–23.49 kg/m^2^	1.21	0.85, 1.71	0.081

a *Complete data available for 1,516 mother-daughter dyads (575 dyads had missing data)*.

This analysis justifies our use of a composite index of maternal capital to explore variability in daughter phenotype, as the effects of maternal capital components on offspring life history trajectory are independent and therefore likely to be cumulative.

### Associations of Maternal Capital With Daughter Traits

Our maternal capital score was expressed as the cumulative number of penalties across the four traits, ranging from 0 to 4. The characteristics of individual maternal traits varied in a dose-response manner in association with the composite score (Table 3). Thus, as expected, the fewer the maternal capital penalties, the greater the mother's height, BMI, income and education. The composite index showed an inverse dose-response association with maternal age (Table 4), but a direct association with the frequency of high parity. Thus, low capital mothers were younger but given their tendency to higher parity, their daughters must on average compete with more siblings for maternal investment.

**Table 3:** Dose-response associations of individual maternal capital traits according to the number of penalties in the composite maternal capital index.

	**Constant**		**1 penalty^$^**		**2 penalties^$^**		**3 penalties^$^**		**4 penalties^$^**		***p*-value^#^**
	**Coeff**	**95%CI**	**Coeff**	**95%CI**	**Coeff**	**95%CI**	**Coeff**	**95%CI**	**Coeff**	**95%CI**	
Maternal height (cm)	163.7	163.1, 164.3	−2.4	−3.2, −1.7	−4.6	−5.4, −3.8	−8.4	−9.3, −7.5	−10.7	−12.2, −9.3	<0.0001
Maternal BMI (kg/m^2^)	24.41	24.07, 24.76	−1.31	−0.88, −1.76	−1.68	−2.13, −1.24	−2.62	−3.14, −2.10	−5.14	−6.03, −4.26	<0.0001
Maternal income (Wages)	7.00	6.46, 7.54	−1.42	−2.11, −0.73	−4.14	−4.84, −3.44	−5.34	−6.16, −4.53	−5.63	−7.01, −4.25	<0.0001
Maternal education (years)	9.74	9.46, 10.02	−2.01	−2.36, −1.65	−4.16	−4.53, −3.80	−5.73	−6.16, −5.31	−6.12	−6.83, −5.40	<0.0001

$ *Each outcome was regressed on four dummy variables, whereby the daughter's mother was identified as having 1, 2, 3, or 4 capital penalties (0 penalties = reference group). The mean coefficient and its 95%CI intervals are shown for each dummy variable. Penalties refer to short stature, low BMI, low education or low family income*.

# *The p-value tests for trend across the maternal capital penalty groups, by regressing each outcome on a single variable coded 0–4 capital penalties. N = 2,005*.

**Table 4:** Dose response associations of maternal or daughter traits according to the number of penalties in maternal capital.

**Predictor**	**0 penalties (*****n*** **=** **389)**		**1 penalty (*****n*** **=** **617)**		**2 penalties (*****n*** **=** **573)**		**3 penalties (*****n*** **=** **299)**		**4 penalties (*****n*** **=** **69)**		***p*-value^a^**
**Maternal traits**	***N***	**%**	***N***	**%**	***N***	**%**	***N***	**%**	***N***	**%**	
First-born	160	41.1	233	37.8	165	28.8	105	35.1	24	34.8	0.001
Fourth^+_^born	21	5.4	56	9.1	76	13.3	40	13.4	10	14.5	<0.0001
Maternal smoking	86	22.1	191	31.0	202	35.3	140	46.8	30	33.3	<0.0001
Maternal alcohol	25	6.4	33	5.3	35	6.1	17	5.7	5	7.2	0.9
	**Coeff**	**95%CI**	**Coeff**	**95%CI**	**Coeff**	**95%CI**	**Coeff**	**95%CI**	**Coeff**	**95%CI**	
Maternal age (y)	27.8	27.2, 28.4	−1.5	−2.3, −0.7	−2.0	−2.7, −1.2	−3.1	−4.1, −2.2	−4.1	−5.7, −2.5	<0.0001
	**Constant**		**1 penalty^$^**		**2 penalties^$^**		**3 penalties^$^**		**4 penalties^$^**		***p*-value^b^**
**Daughter traits**	**Coeff**	**95%CI**	**Coeff**	**95%CI**	**Coeff**	**95%CI**	**Coeff**	**95%CI**	**Coeff**	**95%CI**	
Birth weight (g)	3279	3229, 3329	−110	−174, −47	−214	−279, −149	−301	−376, −225	−453	−582, −325	<0.0001
Birth length (cm)	49.1	48.9, 49.4	−0.6	−0.8, −0.3	−0.8	−1.1, −0.5	−1.3	−1.6, −0.9	−1.6	−2.2, −1.0	<0.0001
Gestational age (w)	38.7	38.5, 38.8	−0.1	−0.3, 0.1	−0.1	−0.3, 0.0	−0.2	−0.4, 0.0	−0.2	−0.5, 0.2	0.015
Excl. breastfed (d)^*^	25.7	19.6, 31.8	−8.6	−16.2, −1.0	−4.8	−12.5, 2.8	−11.3	−20.2, −2.5	−14.7	−28.2, −1.3	0.001
Weight 1 year (kg)^*^	10.1	9.9, 10.3	−0.5	−0.8, −0.2	−0.6	−0.8, −0.3	−0.7	−1.1, −0.4	−1.5	−2.0, −1.0	<0.0001
Length 1 year (cm)^*^	75.4	74.7, 76.5	−1.5	−2.3, −0.7	−1.6	−2.5, −0.8	−2.6	−3.6, −1.7	−3.8	−5.2, −2.3	<0.0001
Age at menarche (y)	12.00	11.88, 12.13	0.01	−0.15, 0.17	0.16	−0.00, 0.32	0.10	−0.09, 0.30	0.52	0.19, 0.84	<0.0001
Height (cm)	163.5	162.8, 164.0	−2.0	−2.8, −1.2	−2.9	−3.7, −2.1	−4.4	−5.3, −3.4	−6.3	−7.9, −4.7	<0.0001
Weight (kg)	64.2	62.9, 65.5	−2.9	−4.5, −1.2	−3.9	−5.5, −2.2	−4.8	−6.8, −2.9	−8.6	−11.9, −5.2	<0.0001
BMI (kg/m^2^)	24.0	23.6, 24.5	−0.5	−1.21, 0.1	−0.6	−1.3, −0.0	−0.6	−1.3, 0.1	−1.5	−2.87, −0.3	0.020
Triceps (mm)	23.6	22.8, 24.5	−1.3	−2.5, −0.2	−2.5	−3.6, −1.3	−2.8	−4.2, −1.5	−4.4	−6.7, −2.1	<0.0001
Subscapular (mm)	17.0	16.2, 17.7	−0.7	−1.7, 0.3	−1.1	−2.1, −0.1	−1.1	−2.2, 0.1	−1.4	−3.3, 0.6	0.028
Fat-free mass (kg)	41.5	41.0, 42.0	−1.1	−1.8, −0.5	−1.5	−2.1, −0.8	−1.9	−2.7, −1.2	−3.8	−5.1, −2.5	<0.0001
Fat mass (kg)	22.7	21.8, 23.6	−1.7	−2.9, −0.6	−2.4	−3.6, −1.2	−2.9	−4.3, −1.5	−4.8	−7.2, −2.3	<0.0001
Glucose (mg/dL)	89.2	87.3, 91.1	0.9	−1.6, 3.3	−0.7	−3.2, 1.7	−0.7	−3.6, 2.2	−0.6	−5.5, 4.4	0.3
Cholesterol (mg/dL)	176.4	173.5, 179.4	−4.9	−8.7, −1.2	−10.4	−14.2, −6.6	−9.7	−14.1, −5.2	−10.2	−17.8, −2.7	<0.0001
HDL (mg/dL)	62.2	61.1, 63.3	−0.6	−2.0, 0.8	−4.1	−5.5, −2.7	−4.8	−6.5, −3.2	−5.7	−8.4, −2.9	<0.0001
LDL (mg/dL)	97.5	95.0, 99.9	−3.8	−6.9, −0.7	−5.4	−8.5, −2.3	−3.7	−7.4, −0.1	−4.5	−10.7, 1.7	0.014
Cholesterol/HDL ratio	2.89	2.83, 2.95	−0.06	−0.13, 0.02	0.02	−0.05, 0.09	0.07	−0.02, 0.15	0.11	−0.04, 0.25	0.007
Triglycerides (mg/dL)	85.3	81.5, 89.0	−3.3	−8.0, 1.4	−6.8	−11.6, −2.0	−9.8	−15.4, −4.2	−6.2	−15.7, 3.3	0.017
HbA1c (%)^¥^	4.83	4.78, 7.90	0.03	−0.04, 0.09	−0.01	−0.08, 0.06	−0.01	−0.09, 0.07	0.13	−0.01, 0.27	0.8
Systolic BP (mmHg)	115.5	114.5, 116.5	−0.1	−1.4, 1.1	−0.7	−2.0, 0.6	−1.2	−2.7, 0.3	−1.3	−3.9, 1.2	0.052
Diastolic BP (mmHg)	70.1	69.2, 70.8	−0.5	−1.5, 0.4	−0.5	−1.5, 0.5	−1.6	−2.7, −0.4	−1.1	−3.1, 0.8	0.018
Education (y)	10.0	9.8, 10.2	−0.4	−0.7, −0.1	−1.3	−1.6, −1.1	−2.0	−2.3, −1.7	−2.5	−3.0, −2.0	<0.0001
Monthly allowance (Rs)	351	307, 395	22	−36, 81	22	−37, 82	65	−8, 139	50	−92, 191	0.14
Gross monthly pay (Rs)	430	396, 464	−14	−57, 29	−11	−55, 32	−13	−63, 37	−34	−117, 48	0.5
	**0 penalties**		**1 penalty**		**2 penalties**		**3 penalties**		**4 penalties**		***p*-value^a^**
	***N***	**%**	***N***	**%**	***N***	**%**	***N***	**%**	***N***	**%**	
Reproduction by 18 y	34	7.0	79	10.4	108	15.7	5	27.0	16	20.5	<0.0001
Studying past year	365	93.8	559	90.6	466	81.5	223	75.3	50	73.5	<0.0001
Studying now	283	72.8	399	64.7	314	54.9	133	44.9	24	35.3	<0.0001
Allowance last month	255	65.6	348	56.4	311	54.4	144	48.6	28	41.2	<0.0001
Pay last year	217	87.9	379	85.6	359	85.5	196	86.0	45	91.8	0.6
Smoked last week	73	18.8	121	19.6	130	22.7	77	26.0	19	27.9	0.068
Current smoker	42	10.8	68	11.0	78	13.6	51	17.2	12	17.6	0.040
Violent crime	19	5.5	39	7.2	58	11.5	23	9.0	6	9.5	0.025

a *p-value chi-square test*.

b *p-value test for trend across the maternal capital groups, by regressing each outcome on a single variable coded 0–4 capital penalties*.

* *Subsample, with analysis adjusted for over-recruitment of low birth weight infants*.

$ *Each outcome was regressed on four dummy variables, whereby the daughter's mother was identified as having 1, 2, 3, or 4 capital penalties (0 penalties = reference group). The mean coefficient and its 95%CI intervals are shown for each dummy variable. Penalties refer to maternal short stature, low BMI < low education or low family income*.

¥ *HbA1c shown as percentage of total hemoglobin. N = 1,947, small level of missing data for body composition and cardio-metabolic outcomes as described in text*.

**Figure 6:**
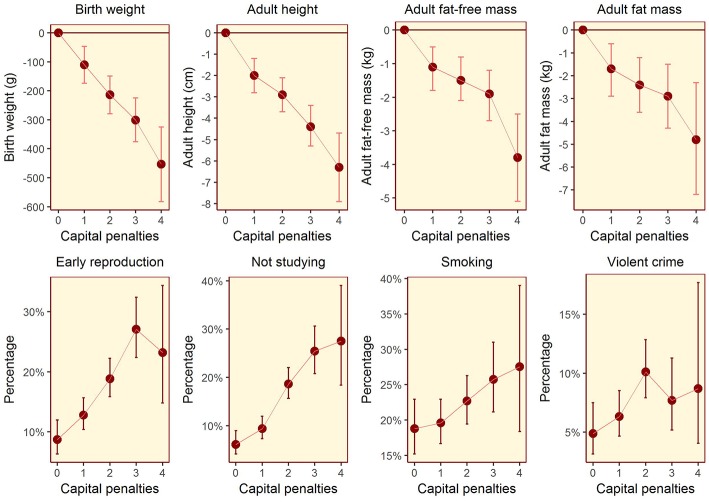
Dose-response associations between daughter traits and the magnitude of maternal capital, categorized in terms of a total composite score of “penalties” (ranging from 0 to 4) selected from the categories “short stature,” “low body mass index,” “low education,” and “low family income.” See text for details of how the index and its categories are defined. The greater the number of capital penalties, the lower the magnitude of maternal capital.

**Figure 7:**
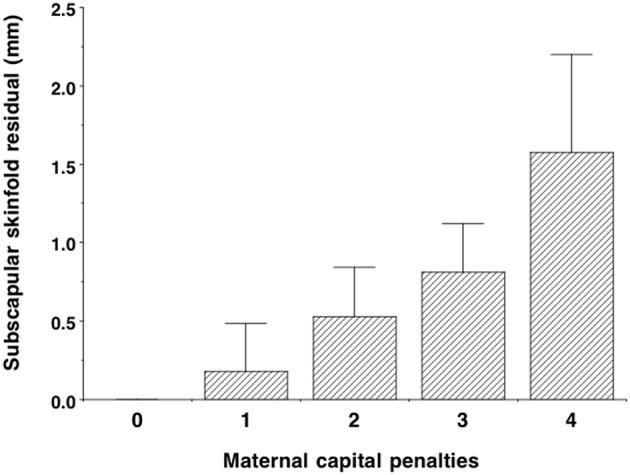
Dose-response associations of subscapular skinfold, adjusted for triceps skinfold, according to the number of “penalties” in maternal capital (ranging from 0 to 4). Lower maternal capital is associated with a more central fat distribution. Test for trend *p* = 0.002.

**Figure 8:**
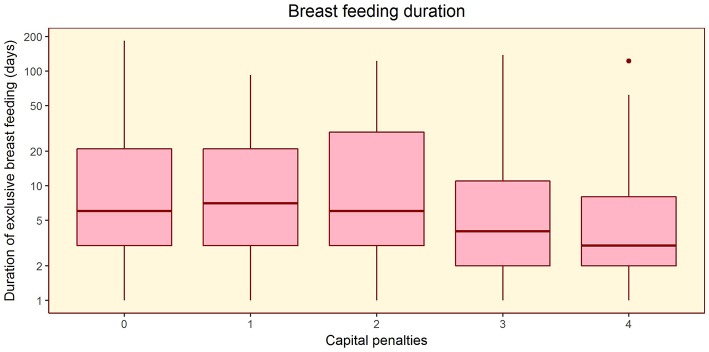
Dose-response associations of the duration of exclusive breast-feeding, according to the number of “penalties” in maternal capital (ranging from 0 to 4). Test for trend *p* = 0.004.

The composite maternal capital index also showed dose-response associations with diverse characteristics of the daughters (Table 4, Figure 6). Of particular importance for our over-arching hypothesis, the greater the number of maternal capital penalties, the greater the likelihood of the daughter reproducing by 18 years, though the highest frequency of early reproduction occurred not in the lowest maternal capital group with 4 penalties (20%), but in the next lowest group with 3 penalties (27%). Broadly, maternal capital was positively associated in dose-response manner with the daughter's growth trajectory and adult size, and the likelihood of her being in school, and inversely with the daughter's likelihood of smoking and of having committed violent crime. Adjusting for triceps skinfold, subscapular skinfold increased in association with the number of maternal capital penalties (*p* = 0.002), indicating a more central fat distribution in daughters receiving low maternal investment (Figure 7). However, associations with cardio-metabolic risk markers with the maternal capital index were inconsistent. The duration of exclusive breast-feeding fell with increasing number of maternal capital penalties (test for trend *p* < 0.004), but the magnitude of this effect was very small (Figure 8).

Figure 3B (see Supplementary Table 2 for numerical values) illustrates these patterns by contrasting the daughters from the highest and lowest maternal capital groups. The results show substantial similarity with the comparison of daughters with/without offspring, as shown in the Figure 3A. The low and high maternal groups contrasted strongly in the individual maternal traits: low-capital mothers had substantially lower values for mean height (Δ = 10.7 cm, 95%CI 9.9, 11.5), pre-pregnancy BMI (Δ = 5.1 kg/m^2^, 95%CI 4.7, 5.6), educational attainment (Δ = 6.1 y, 95%CI 5.6, 6.6), and family income (Δ = 5.6 minimum wages, 95%CI 5.0, 6.2). Low-capital mothers were more than twice as likely to smoke during pregnancy, but no more likely to drink alcohol. Low-capital mothers were nearly three times more likely than high-capital mothers to be high-parity (4^+^ offspring), but were similarly likely to be first-time mothers. Daughters of low-capital mothers were 3.2 times as likely as those of high-capital mothers to have reproduced by 18 years, and 8.6 (95%CI 1.4, 52.6) times more likely to have two or more children by this age.

Daughters of low-capital mothers were lighter and shorter at birth and remained smaller at 1 year than those of high-capital mothers, and achieved menarche 5 months later. Low-capital daughters remained 6.3 cm shorter and 1.2 kg lighter at 18 years, and had lower lean body mass and whole-body fat mass. They had lower triceps but similar subscapular skinfold, indicating a more central abdominal fat distribution. Taking into account the average age at menarche and adult height of each group, we estimate that the daughters of low-capital mothers would on average have been shorter and lighter than those of high-capital mothers at the time of menarche.

Daughters of low-capital mothers did not differ from those of high-capital mothers in their fasting glucose, triglycerides or blood pressure. They had lower total cholesterol, with this difference due to significantly lower HDL (“healthy” cholesterol) whereas “unhealthy” LDL cholesterol was similar between the groups. Thus, in contrast to the associations with body size and body composition, maternal capital was not a good predictor of daughters' cardio-metabolic physiology.

Daughters of low-capital mothers were only one fifth as likely as high-capital daughters to have studied over the previous year, or to be in school at the time of the follow-up, and on average they had completed 2.5 (95%CI 2.0, 2.9) fewer years education. Low-capital daughters were less likely to have received a monthly allowance in the previous month, though if they did it was of similar magnitude to that received by high-capital daughters (Δ = −40 Rs, 95%CI −142, 63). They were equally likely to have been paid for work, and for those working, the monthly payment was similar to that received by high-capital daughters (Δ = 32 Rs, 95%CI −38, 101). Low-capital daughters were almost twice as likely to smoke, and more than twice as likely to have committed violent crime.

Overall, lower maternal capital was associated with an increased risk of early reproduction in the daughter, along with poorer linear growth, less healthy fat distribution, lower educational attainment, and greater likelihood of risky behavior.

### Clustering of Outcomes and Interaction Analysis

We considered two possible drivers of clustering of adverse outcomes in the daughters: early reproduction and low maternal capital. First, we compared all daughters who had reproduced by 18 years against those who had not. Daughters who reproduced by 18 years comprised 14.8% of the population, but accounted for 18.4% of obesity, 20.0% of violent crime, 20.3% of short stature, 32.2% of current smoking and 52.0% of school dropout. They also accounted for 20.2% of low birth weight.

In turn, we combined the daughters from the two groups characterized by the lowest levels of maternal capital (either 3 or 4 penalties, *n* = 359), and compared this group against the remainder of the cohort. The low capital group comprised 18.6% of the population of daughters, and accounted for 17.5% of obesity, 19.6% of violent crime, 24.9% of smoking, 27.7% of short stature, 28.6% of low birth weight, 32.1% of school dropout, and 34.1% of early reproduction. Thus, the most common adverse outcome in the low capital segment was early childbearing.

The interactive associations of adverse outcomes with daughter's exposure to low maternal capital and early reproduction are given in Table 5. Compared to simple models, which compared the outcomes either across the low vs. high maternal capital groups, or between early reproducing daughters vs. those not reproducing, the model that compared across all four groups explained more variability in the distribution, indicated by higher likelihood ratio values. For most traits, the pattern was very similar: among early reproducing daughters, outcomes were worse if her mother was also in the low capital group, compared to the high capital group. Similarly, among daughters with low maternal capital, outcomes were worse if the daughter had also reproduced early, compared to not reproducing early. Thus, the worst outcomes for almost every trait were evident in daughters with both low maternal capital and early reproduction, however the one exception was overweight, where the worst outcome was evident in early reproducing daughters from the high maternal capital group.

**Table 5:** Chi-squared tests of interactive associations of maternal capital status and daughter reproductive status with adverse daughter outcomes.

**Early reproduction**	**No**		**Yes**		**Chi-squared tests**					
**Maternal capital**	**High (*n* = 371)**	**Low (*n* = 284)**	**High (*n* = 28)**	**Low (*n* = 97)**	**Maternal capital**		**Daughter reproduction**		**All 4 groups**	
	**Group A**	**Group B**	**Group C**	**Group D**	**(A+C) vs. (B+D)**		**(A+B) vs. (C+D)**		**A vs. B vs. C vs. D**	
	**%**	**%**	**%**	**%**	**LR**	***p*-value**	**LR**	***p*-value**	**LR**	***p*-value**
Studying last year	96.2	85.9	60.7	43.3	54.9	<0.0001	123.9	0.0001	149.3	<0.0001
Studying now	75.2	53.5	28.6	13.4	66.5	<0.0001	106.9	0.0001	143.7	<0.0001
Receiving support	65.5	52.1	64.3	29.9	28.6	<0.0001	20.7	0.0001	43.3	<0.0001
Paid for work	88.9	88.8	80.0	77.9	0.6	0.4	6.9	0.005	6.9	0.050
Smoked last week	18.6	19.7	25.0	48.5	7.0	0.008	30.8	0.0001	36.0	<0.0001
Current smoker	10.0	11.3	17.9	33.0	6.5	0.011	27.1	0.0001	30.0	<0.0001
Low birth weight	5.9	16.5	7.1	14.4	20.6	<0.0001	0.5	0.4	20.9	<0.0001
Overweight	31.2	25.9	60.0	31.5	2.9	0.086	3.4	0.061	12.1	0.004
Short stature	13.3	35.2	12.0	46.1	62.0	<0.0001	12.1	<0.0001	65.4	<0.0001
Violent crime	5.5	10.7	8.7	4.9	3.3	0.071	0.5	0.4	6.4	0.089

*LR, likelihood ratio value from chi-squared test. N = 780*.

Both early reproduction and low maternal capital therefore contribute to clustering of adverse outcomes among individuals in this population, and they may represent different components of the overall causal chain. Overall, the worst outcomes occurred among daughters characterized by both early reproduction and exposure to low maternal capital in early life.

### Adjusting for Direct Mother-Daughter Transmission of Traits

Given that early-reproducing daughters were both shorter, and also the offspring of shorter mothers, maternal phenotype should be controlled for when testing the association of early reproduction with the daughter's growth trajectory. Such associations might be driven by shared genetic factors, or non-genetic inter-generational transmission. A similar scenario applies to behavioral traits, which might be replicated across generations due to shared family habits or opportunities.

Adjusting for maternal height, length at birth and 1 year and adult height all remained inversely associated with the odds of early reproduction by the daughter (Table 6). Conversely, adjusting for maternal size, low weight at birth and 1 year was inversely associated with the odds of early reproduction by the daughter, whereas weight, BMI, and fat mass index at 18 years were positively associated with the risk. Of particular interest, the model for BMI showed contrasting associations between the mother and daughter, with lower maternal BMI, and higher daughter BMI, predicting early reproduction by the daughter. Overall, these associations indicated that poor early growth followed by greater gains in weight and fat after 1 year favored early reproduction, indicating an overall growth trajectory favoring weight gain over linear growth, as indicated in Figure 4. Moreover, the association between early reproduction and short adult stature was primarily driven not by reproduction curtailing adolescent growth, but rather by poor early growth patterns predicting early reproduction.

**Table 6:** Odds ratio of early reproduction associated with the daughter's growth phenotype, without/with adjustment for maternal phenotype.

**Predictor**	**Model 1 (unadjusted)**		**Model 2 (adjusted)**	
	**Odds ratio**	**95%CI**	**Odds ratio**	**95%CI**
Birth weight (kg)	0.579	0.458, 0.733	0.618	0.482, 0.793
Maternal weight (kg)			0.985	0.972, 0.999
Weight 1 y (kg)	0.820	0.682, 0.985	0.855	0.690, 1.059
Maternal weight (kg)			1.008	0.984, 1.032
Weight 18 y (kg)	0.842	0.688, 1.030	1.013	1.003, 1.024
Maternal weight (kg)			0.973	0.959, 0.987
Birth length (cm)	0.927	0.882, 0.975	0.941	0.894, 0.992
Maternal height (cm)			0.956	0.938, 0.975
Length 1 y (cm)	0.864	0.799, 0.934	0.872	0.804, 0.945
Maternal height (cm)			0.985	0.949, 1.022
Height 18 y (cm)	0.939	0.920, 0.958	0.948	0.927, 0.969
Maternal height (cm)			0.977	0.957, 0.999
Birth BMI (kg/m^2^)	0.866	0.796, 0.941	0.841	0.798, 0.949
Maternal BMI (kg/m^2^)			0.991	0.957, 1.026
BMI 18 y (kg/m^2^)	1.043	1.018, 1.069	1.057	1.030, 1.085
Maternal BMI (kg/m^2^)			0.954	0.918, 0.991
Fat mass index 18 y (kg/m^2^)	1.032	0.998, 1.067	1.047	1.011, 1.085
Maternal BMI (kg/m^2^)			0.962	0.926, 0.999

*BMI, body mass index. N = 2,071, except for weight and length at 1 year where n = 594*.

Likewise, adjusting for the equivalent component of maternal phenotype, early reproducing daughters had attained fewer years of education, were less likely to be in school, and more likely to smoke (Table 7).

**Table 7:** Association of early reproduction with components of daughter phenotype, without/with adjustment for the same, or similar component of maternal phenotype.

**Outcome in daughter**	**Predictor**	**Model 1 (unadjusted)**		**Model 2 (adjusted)**	
		Coeff	95%CI	Coeff	95%CI
Years of education	Early reproduction	−2.44	−2.68, −2.21	−1.96	−2.18, −1.74
	Maternal education (y)			0.23	0.21, 0.25
		OR	95%CI	OR	95%CI
Studying now	Early reproduction	0.10	0.07, 0.13	0.11	0.09, 0.15
	Maternal education 1			0.31	0.22, 0.46
	Maternal education 2			0.35	0.24, 0.50
Current smoker	Early reproduction	3.44	2.57, 4.59	3.10	2.31, 4.16
	Maternal smoking			2.14	1.64, 2.78

*Maternal education group 1: 0–4 years, group 2: 5–7 years; reference group 8+ years. Maternal income group 1: 0–2 minimum wages, group 2: 3–4 minimum wages, reference group 5+ minimum wages. N = 2,091*.

In general, therefore, early reproduction was associated with unfavorable trade-offs relating to growth, health, education and risk-taking, independent of the potential direct maternal transmission of phenotypic traits.

## Discussion

The early developmental period, during which phenotype is most sensitive to the environment, is also the period when environmental stresses and stimuli are primarily transduced through maternal phenotype (20, 41). On this basis, we hypothesized that maternal capital, a marker of the resources available for investment, may leave a unique imprint on the offspring during early life, impacting both behavioral and physiological traits. Specifically, we predicted that early reproduction in daughters would correlate with risk-prone behavior, and demonstrate trade-offs with each of growth, health, and investment in education. We further predicted that such trade-offs would be induced by exposure to low maternal capital in early life. Our results support this conceptual approach, with several key findings.

First, supporting Figure 2A, we found that early reproduction was indeed associated with reduced investment in competing traits, including adult height and lean mass, and levels of education. Early reproduction was also associated with a greater propensity for risky behavior, and with some markers of poorer cardio-metabolic health, such as a higher risk of obesity, more central fat deposition and a less favorable cholesterol profile.

Second, these trade-offs began to emerge early in development, indicated by contrasting growth trajectories. On average, early-reproducing daughters were smaller at birth than their childless peers and remained shorter in adulthood, but by 1 year of age they had negated their deficit in weight, and by adulthood they demonstrated higher BMI and markers of whole-body and central adiposity. These growth contrasts were not mediated by age at menarche, rather the significant factor was a trade-off between linear growth and weight gain, commencing in early life. On this basis, the trajectory to early reproduction had already started before birth, and any increase in nutritional supply in early post-natal life did not resolve earlier deficits in linear growth, but rather promoted weight gain. A more extreme version of this scenario is illustrated by international adoption studies, which have found that if poor early growth is followed by exposure to increased resources in childhood, sexual maturation is substantially accelerated without promoting linear growth (42, 43).

Third, supporting Figure 2B, early reproduction was associated with low maternal capital, with early-reproducing daughters born to younger, shorter, poorer, and less educated mothers. Their mothers were also more likely to smoke and to have larger family size, indicative of greater inter-sibling competition for maternal investment. The importance of maternal investment for explaining variability in daughter phenotype was confirmed by comparing groups of daughters stratified by high or low levels of our composite index of maternal capital. Supporting Figure 2C, low maternal capital was associated with the daughter trading off growth and education in favor of greater likelihood of early reproduction, central adiposity, and risky behaviors. Again, these patterns were initiated in early life, demonstrated by reduced birth weight and infant growth. Menarche occurred later in the low capital daughters, despite their higher odds of reproducing by 18 years, indicating continued constraint of linear growth. Sexual maturation therefore appears to have occurred at smaller body size rather than younger age in the daughters of low capital mothers, consistent with other studies in low-/middle-income countries (44, 45).

Fourth, an important finding was that early-reproducing daughters did not simply replicate maternal traits. Rather, holding constant the presence or absence of a particular adverse outcome in the mother, markers of poor growth, and development in the daughter were independent predictors of early reproduction by the daughter. Likewise, early reproduction by the daughter was associated with lower educational attainment and current smoking, independent of the mother's education and smoking status at baseline. An intriguing finding was that lower maternal BMI, but higher daughter BMI, predicted early reproduction by the daughter.

Fifth, supporting Figure 2D, we found a synergistic association of adverse outcomes with exposure to low maternal capital and early reproduction. Specifically, outcomes were worse among low-capital daughters if they had also reproduced early, while outcomes were worse among early reproducing daughters if they had also been exposed to low maternal capital.

These trade-offs indicate a generic link between low maternal investment and “future discounting” by the offspring that is both mediated by, and also exacerbated by, early reproduction. These findings are consistent with other studies linking poor early growth with lower educational attainment (46–48), earlier reproduction (49), reduced family income (50), and poorer adult health (51, 52). These associations furthermore demonstrate a dose-response nature, so that the greater the magnitude of maternal capital, the better the daughter's growth and educational progress, and the lower the likelihood of her reproducing by 18 years.

However, an interesting finding was that the highest frequency of early childbearing occurred not among daughters in the lowest maternal capital group, but in the second-lowest group. We speculate that the daughters of the lowest capital mothers experienced continued exposure to poverty during childhood, which may have constrained the magnitude of the resulting trade-offs, including the likelihood of reproducing early. Consistent with that hypothesis, they had a later age of menarche than all the other groups (Table 4). Although low birth weight has been associated with earlier menarche in high-income populations, the magnitude of this effect is very small (difference of 2 months compared to high birth weights), whereas daughters with higher weight at 7 years underwent menarche a full year earlier compared with those with low weight at this age (53). There may be human societies in which access to resources during development is so constrained among those of lower rank that higher-ranking individuals reproduce earlier, and this scenario has been reported in several primate species (20). Of relevance to understanding such variability in human populations, age at menarche in low-middle-income countries correlates with infant mortality rate (54), suggesting that when a greater proportion of energy intake is diverted to immune function the pace of maturation is reduced.

Fifth, we showed that the segments of the population reproducing early, or exposed to low maternal capital, were characterized by a cluster of adverse outcomes including shorter stature, more central fat deposition, less education, and more risky behavior. The evidence for a trade-off between early reproduction and health markers was weaker, however this is most likely because non-communicable diseases primarily develop later in the adult life-course. Early-reproducing daughters had few overt markers of cardio-metabolic risk, but their lower birth weight, shorter stature, higher BMI, and more central fat distribution all predict poorer cardio-metabolic health from middle age onwards. As mentioned in the introduction, the closer association of abdominal body fat than peripheral fat with markers of immune function allows us to consider variability in central adiposity as a broad marker of investment in immune function (28). Our results therefore indicate that daughters exposed to low maternal capital prioritized the allocation of energy to immune defense, which may help resist infections in the short term, but at a cost to long-term cardio-metabolic health.

Our results offer unique insight into why inter-generational cycles of disadvantage are perpetuated: exposure to lower maternal capital is associated with a life history trajectory that compromises physical and educational development, in favor of an increased susceptibility to early reproduction, accompanied by greater immune defense. Those who realize this tendency for early reproduction compound these trade-offs. The capacity for facultative adjustment of life history strategy in response to maternal capital has been predicted to be crucial in hierarchical societies, due to the long-term consequences of variability in early growth trajectories (23).

Our findings are consistent with previous research, which has linked parental investment with the life-history trajectory of offspring, especially daughters. For example, studies on US children have linked the timing of menarche with the quality of mother-child relationships (18), or markers of paternal investment (44). However, few studies have extended this approach to markers of pre-natal maternal investment. Exceptions include a study of young South Asian women living in the UK, which found that those with lower birth weight (indicating reduced maternal investment *in utero*) were characterized by trade-offs favoring reproductive potential over growth and the maintenance of health (55). In a larger UK birth cohort, low birth weight was associated with faster maturation and earlier anticipation of starting a family during adolescence, followed by increased likelihood of reproducing before 20 years (49). Overall, such research remains largely limited to high-income western populations, and there is need for studies in other settings, and to address male offspring (56).

Both the behavioral and physiological trade-offs we have documented are consistent with the logic of future discounting. A link between early reproduction with social or economic disadvantage during early life is well-recognized (57), as are associations in adulthood of early childbearing with poor health outcomes and reduced life opportunities (30, 31). Likewise, previous research using similar conceptual models has linked higher perceived extrinsic mortality risk with higher rates of avoidable death at the population level, and with less effort in maintaining health and earlier reproduction at the individual level (19, 58). In terms of behavior, those whose family members typically perform low-skilled work may see less value in extended education, since it delays access to wages (59), while high community rates of adult violence and morbidity may negate public health advice to preserve health by avoiding smoking or high alcohol consumption (58). Although markers of adversity during development seem to be universally associated with riskier behavior, the exact nature of the risky behavior undertaken may differ between populations according to cultural norms, which may account for our null findings for maternal alcohol consumption.

By the same logic, poor growth in early life reduces the constitutional somatic quality of the body, through depleting components of organ function that contribute to homeostasis. As encapsulated in the “disposable soma” hypothesis, the payoff for investing in “maintenance” to reduce the rate of deterioration in old age must inevitably decline, if the chances of reaching old age are reduced (7, 13). This may help explain the elevated diabetes susceptibility in old age among those with low birth weight, since investing in the capacity to prevent diabetes in the long-term will benefit only a minority of such individuals if the majority are likely to succumb to mortality earlier (7, 60). Our analysis builds on these earlier studies, by linking early reproduction with both physiological and behavioral outcomes indicative of future discounting. Our key new insight is that this future discounting is associated with low maternal investment.

A variety of theoretical frameworks have been used to study the consequences of exposure to adversity and deprived environments on markers of health and human capital (61, 62). A very simple conceptual approach, for example, could predict that adverse outcomes are inter-correlated, and that those at the lowest level of social hierarchies tend to have less of everything. However, our findings do not support such a simple model—it is not clear, for example, why mothers with low capital should have daughters who are more likely to smoke (a habit that incurs regular financial costs) or reproduce early (which also generates metabolic and material costs). Life history theory offers a better fit with these findings, through its incorporation of the principles of trade-offs and future discounting. In a different vein, there are also other theoretical frameworks that are not necessarily inconsistent with life history theory, and which make similar predictions based on different theoretical assumptions (62). It is already recognized that different theories may be addressing proximate or ultimate levels of explanation, and that they may be complementary rather than contradictory (62).

In identifying trade-offs, it is important not to blame mothers for their low capital, or for the adverse outcomes in their daughters, or indeed the daughters themselves. As indicated by our evidence on inter-generational associations, being short, thin, uneducated, and poor are traits that are largely beyond individual control. It is noteworthy that relevant underlying trade-offs were already emerging during fetal life. Interestingly, maternal capital status was only weakly correlated with the duration of exclusive breast-feeding, where mothers may have greater opportunity consciously to influence their investment in their offspring. Early reproduction is widely considered an unsatisfactory trait by policy makers. However, a tendency to reproduce early in adverse environments is expected to have been favored by selection in ancestral environments, in the face of high extrinsic mortality risk. Such trade-offs may continue to be elicited in contemporary environments, even though they adversely impact health and educational attainment in both generations. The emphasis should therefore be on improving environmental conditions, to reduce these trade-offs.

### Strengths and Limitations

Among the strengths of the study are the prospective data on maternal capital and early growth patterns of the daughters, the wide range of outcomes spanning both physiology and behavior assessed in the daughters, the large sample size, and the relatively high cohort retention rate at 18 years. Moreover, very few studies have previously tested life history predictions in low-/middle-income country populations, in particular those characterized by very high levels of social inequality.

Our study also had several limitations. First, a fifth of the cohort did not participate in the adult follow-up, however baseline differences between those followed and those not followed were few and of small magnitude, hence any bias from this source is unlikely to jeopardize our findings. The lower average weight and length at birth among those not followed up suggests that the magnitude of the association of poor growth with elevated risk of early reproduction we report may be conservative. However, the only maternal trait that varied by follow-up status was BMI, which showed no independent association with early reproduction by the daughter. Hence there is likely to be negligible bias in our overall assessment of the association between maternal capital and adverse daughter outcomes.

Second, a small amount of data was missing for both maternal and daughter traits, in most cases <2% but reaching a maximum of 11.7% for the violent crime outcome in daughters. However, we consider that these missing data had little effect on our results. For the mothers, the missing data were imputed, but the findings changed negligibly. For the daughters, those missing data on anthropometry and body composition tended to have shorter, younger and less educated mothers, while those missing data on violent crime had mothers with lower family income, but in each case the magnitude of these differences relative to the range in the entire population was small. Data on the blood test appeared to be missing at random.

Third, we elected to use a categorical approach to integrate the different components of maternal phenotype, rather than conducting analysis of continuous variables. This approach reduced our ability to assess daughter outcomes across the whole range of maternal capital, however the benefit was that it allowed us to focus on mothers who were in the bottom tertile of the population for any given trait, i.e., a high-risk group. Moreover, by counting “maternal capital penalties,” we could analyse the consequences of mothers lacking capital across a range of different outcomes. We used the same categorical approach for daughters, in order to assess the odds of daughters falling into high-risk groups. Overall, we suggest that this focus on high-risk groups among both mothers and daughters helps understand inter-generational cycles of disadvantage. While different approaches might have emphasized different associations in these data, we believe that our findings are robust and would have broadly emerged using other analytical designs.

Fourth, we lacked data on paternal phenotype (education, income, height, nutritional status, presence in the household) and therefore could not test for independent effects of paternal investment. Although we treated household income as a component of maternal capital, it may commonly relate to both parents' earning capacity. Similarly, education is characterized by assortative mating, with men tending to marry women of equal or fewer years of schooling, hence we cannot differentiate a specific association attributable to the mother's education. If height of the two parents is correlated, then likewise, the reduced early growth rates of offspring of shorter mothers might in part reflect a low paternal growth drive. However, while acknowledging these paternal effects, we could also consider such assortative mating with low capital fathers to represent the “extended phenotype” of the low-capital mother (63), since high-capital mothers are likely to use their phenotype (greater wealth, education, and body size) to increase their chances of pairing with high-capital fathers.

Fifth, we had information on only a single child within each family, and could not therefore test how maternal investment might vary through the reproductive career. The fact that low-capital mothers tended to have higher parity than high-mothers suggests greater competition between the offspring of low capital mothers for a smaller pool of maternal resources. Since mothers may accumulate capital through the life-course, and since low capital mothers tend as we have shown to reproduce early, the adverse consequences for the offspring of low capital mothers might be greatest if they are also first-borns. Given the birth order differences in our sample, our findings may therefore be conservative. More generally our findings relate to a specific time period and specific city in southern Brazil, and it is unclear how they might generalize to other periods or geographical settings.

Sixth, our analysis was observational, and could not demonstrate causal associations of maternal capital and daughter outcomes, nor direct trade-offs between the different daughter outcomes. While correlations between life history traits are widely interpreted as demonstrating trade-offs in human studies (64–66), an experimental approach would be necessary to demonstrate that increasing or decreasing energy allocation to one function causes the opposite effect on another function. However, mathematical modeling offers another opportunity to provide support for our interpretation (67).

Future work in this area could extend our approach to male offspring, and could use alternative approaches to derive cumulative maternal capital scores. Regarding outcomes, it would be valuable to assess more direct markers of immune function such as leptin, or the status of pro-inflammatory markers. Finally, while our focus here was on the manifestation of trade-offs at the onset of adulthood when aging effects are modest, longer-term follow-up would allow us to investigate how these trade-offs might change across the daughter's reproductive career. In addition, it would be possible investigate how daughters' lifetime fitness itself varies in association with the timing of reproduction and the magnitude of maternal capital to which they were exposed in early life.

In conclusion, our study has shown that early reproduction is associated with an increased likelihood of adverse adult outcomes, which are expected to impact adversely on future generations. In this way, we highlight the central role of trade-offs associated with early reproduction in perpetuating inter-generational cycles of disadvantage. We further showed that the life history trajectory and adult phenotype of daughters can be predicted from a composite index of maternal capital, with both physical and socio-economic maternal traits contributing. Both behavioral and physiological outcomes demonstrated a common pattern of future discounting.

Our life history approach helps understand why some segments of a population may contribute disproportionately to traits that generate a large economic burden on the population (1). We explained less clustering than Caspi et al., but this may be due to our focus on early reproduction to assess adult trade-offs, and to the relatively young age of the sample (18 years, compared to 38 years in the Dunedin cohort). Clustering may become more pronounced with age, particularly regarding health markers. Although post-natal factors affecting adult outcomes are also relevant, our study is important in showing that factors acting in prenatal life and infancy already account for some clustering of adverse outcomes.

Differences in female growth up to age 18 had largely been determined by the end of infancy in our study, while other studies indicate that secular trends in height are also largely accounted for by growth prior to 2 years (67). The policy implications are that interventions targeting childhood and adolescence are likely to come too late to resolve these high-risk developmental trajectories. While efforts to prevent early reproduction will reduce the consequences of exposure to low maternal capital, the most effective intervention to improve daughter outcomes would be to improve maternal capital, thus reducing the risks of poor early growth and early reproduction in the daughter. Interventions on both health and education are likely to achieve their greatest beneficial effects if they target women before they conceive their offspring. Moreover, interventions should be sustained over a multi-generational time-frame for the full benefits to emerge, through the cumulative exposure of each generation to favorable maternal capital during early critical windows. Finally, societies need to become more gender equal so women have equal access to resources to accumulate their maternal capital. Such an approach is likely to be essential to resolve inter-generational cycles of disadvantage that adversely affect both men and women. The incentive for such an approach is that successful interventions could produce benefits across a wide range of behavioral and physiological traits.

## Data Availability

The datasets for this manuscript are not publicly available because the data on this longitudinal birth cohort are curated by the Federal University of Pelotas and must safe-guard participant privacy. The dataset analyzed during the current study is available from the corresponding author on reasonable request. Requests to access the datasets should be directed to jonathan.wells@ucl.ac.uk.

## Ethics Statement

In all phases of the study, ethical approval was obtained from the Medical School Ethics Committee of the Federal University of Pelotas and full informed consent was provided by parents or their legal representatives (if the subject was aged under 18 years) or by cohort members. Verbal consent was provided in the perinatal phase.

## Author Contributions

JW, TC, MC-B, and AMa designed the analysis. The Pelotas 1993 birth cohort is overseen by AMe, who steered the 18-year follow-up conducted by FW, PO, HG, and IO. Initial statistical analysis was undertaken by JW, TC, and MC-B, and revised in light of feedback from AMa, JM, AMe, RS, and DL. JW and MC-B developed the composite Figure 2. All authors reviewed drafts, provided critical feedback, and approved the final manuscript.

### Conflict of Interest Statement

The authors declare that the research was conducted in the absence of any commercial or financial relationships that could be construed as a potential conflict of interest.

## Footnotes

^1^Atlas of Human Development in Brazil.
