# Supplementary material for: Low Maternal Capital Predicts Life History Trade-Offs in Daughters: Why Adverse Outcomes Cluster in Individuals
Source: Front Public Health. 2019 Jul 31;7:206. doi: 10.3389/fpubh.2019.00206 (PMC6685417; doi:10.3389/fpubh.2019.00206)
Supplement: Supplementary file 1 [file Table_1.pdf]

Supplementary online material for

Low maternal capital predicts life history trade-offs in daughters: why adverse outcomes cluster in individuals

Jonathan CK Wells, Tim J Cole, Mario Cortina-Borja, Rebecca Sear, David A Leon, Akanksha A Marphatia, Joseph Murray, Fernando César Wehrmeister, Paula Duarte Oliveira, Helen Gonçalves, Isabel O. Oliveira, Ana Maria Baptista Menezes

Supplementary online Table 1. Differences between those followed up versus those not followed

	**Followed up (*n*=2091)**		**Not followed (*n*=554)**				
**Maternal characteristic**	N	%	N	%	OR*	95% CI	*p*
Parity 0 [$]	736	35.2	188	33.9	1.06	0.87, 1.29	0.5
Parity 4^{+} [$]	221	10.6	66	11.9	0.87	0.65, 1.17	0.3
Smoking in pregnancy [$]	705	33.7	204	36.8	0.87	0.72, 1.06	0.17
Alcohol in pregnancy [$]	122	5.8	22	4.0	0.67	0.42, 1.06	0.086
	Mean	SD	Mean	SD	Difference*	95% CI	*p*
Maternal age (y) [#]	26.1	6.3	25.8	6.6	0.3	-0.3, 0.9	0.3
Maternal height (cm) [#]	159.9	6.7	159.4	7.1	0.5	-0.1, 1.1	0.13
Maternal BMI (kg/m^2) [#]	22.9	3.7	22.4	3.7	0.4	0.1, 0.8	0.013
Maternal education (y) [#]	6.7	3.5	6.5	4.0	0.2	-0.1, 0.6	0.2
Family income (minimum wages) [#]	4.3	5.8	4.4	5.9	-0.1	-0.7, 0.4	0.6
**Daughter characteristic**	Mean	SD	Mean	SD	Difference*	95% CI	*p*
Birth weight (g)	3115	510	3012	578	-103	-53, -152	<0.0001
Birth length (cm)	48.5	2.3	48.2	2.3	-0.3	-0.1, -0.5	0.013
Gestational age (weeks)	38.6	1.5	38.3	1.7	-0.3	-0.1, 0.4	0.001

* OR and difference values are calculated for those not followed up, relative to those followed up

[$] Categorical outcomes compared by Chi-square test

[#] Continuous outcomes tested by two-sample t-test
