# Supplementary material for: Low Maternal Capital Predicts Life History Trade-Offs in Daughters: Why Adverse Outcomes Cluster in Individuals
Source: Front Public Health. 2019 Jul 31;7:206. doi: 10.3389/fpubh.2019.00206 (PMC6685417; doi:10.3389/fpubh.2019.00206)
Supplement: Supplementary file 2 [file Table_2.pdf]

Supplementary online material for

Low maternal capital predicts life history trade-offs in daughters: why adverse outcomes cluster in individuals

Jonathan CK Wells, Tim J Cole, Mario Cortina-Borja, Rebecca Sear, David A Leon, Akanksha A Marphatia, Joseph Murray, Fernando César Wehrmeister, Paula Duarte Oliveira, Helen Gonçalves, Isabel O. Oliveira, Ana Maria Baptista Menezes

Supplementary online Table 2. Numeric results for comparisons of (a) reproducing vs non-reproducing daughters and (b) low-capital vs high-capital mothers

	Data for Figure 2 Panel A<br>**Early-reproducing daughters, relative to childless peers [1]**		Data for Figure 2 Panel B<br>**Daughters of low-capital mothers, relative to those of high-capital mothers [2]**	
Trait				
**Pregnancy traits**	**OR**	**95%CI**	**OR**	**95%CI**
Parity 0	0.63	0.48, 0.83	0.77	0.46, 1.30
Parity 4$^+$	1.66	1.17, 2.35	2.90	1.30, 6.46
Smoking in pregnancy	1.99	1.56, 2.54	2.52	1.49, 4.27
Alcohol in pregnancy	0.78	0.48, 1.25	0.93	0.35, 2.52
**Developmental traits**	**% difference**	**95%CI**	**% difference**	**95%CI**
Birth weight (g)	-4.7	-6.8, -2.6	-13.7	-17.7, -9.6
Birth length (cm)	-0.8	-1.4, -0.2	-3.1	-4.2, -2.0
Gestational age (weeks)	-0.5	-1.0, -0.0	-0.3	-1.2, 0.5
Weight 1 year (kg)	-1.9	-4.9, 1.1	-16.0	-21.0, -10.9
Length 1 year (cm)	-1.8	-2.9, -0.8	-5.2	-7.0, -3.4
Age at menarche (y)	-0.4	-1.8, 1.0	4.4	1.8, 6.9
**Adult physical traits**	**% difference**	**95%CI**	**% difference**	**95%CI**
Height (cm)	-1.6	-2.1, -1.1	-3.9	-4.9, -2.9
Weight (kg)	1.3	-1.2, 3.9	-14.6	-19.6, -9.5
BMI (kg/m^2)	4.5	2.1, 6.8	-6.8	-11.7, -1.9
Fat-free mass (kg)	0.9	-0.7, 2.5	-9.6	-12.7, -6.6

Fat mass (kg)	1.8	-3.5, 7.1	-26.3	-36.9, -15.7
Fat mass index (kg/m^2)	4.9	-0.3, 10.2	-18.6	-28.4, -8.7
Triceps skinfold (mm)	-7.7	-12.8, -2.5	-24.5	-36.4, -12.6
Subscapular (mm)	5.0	-0.6, 10.6	-10.0	-21.2, 1.1
Glucose (mg/dL)	-0.6	-2.8, 1.6	-0.3	-4.4, 3.8
Cholesterol (mg/dL)	-3.0	-5.1, -0.8	-5.8	-10.3, -1.2
HDL (mg/dL)	-8.7	-11.0, -6.4	-9.3	-14.1, -4.5
LDL (mg/dL)	-0.2	-3.3, 3.0	-5.0	-11.7, 1.8
Triglycerides (mg/dL)	-3.7	-8.5, 1.0	-8.0	-18.8, 2.8
HbA1c (%)	-0.6	-1.9, 0.7	2.5	0.1, 5.1
Systolic BP (mmHG)	-0.3	-1.4, 0.8	-1.2	-3.6, 1.1
Diastolic BP (mmHG)	-2.2	-3.8, -0.7	-1.7	-4.6, 1.2
	**OR**	**95%CI**	**OR**	**95%CI**
Studying past year	0.10	0.07, 0.13	0.20	0.10, 0.39
Studying now	0.10	0.07, 0.14	0.22	0.13, 0.37
Received allowance in last month	0.42	0.32, 0.53	0.36	0.21, 0.62
Received payment for work in last year	0.45	0.32, 0.63	1.59	0.53, 4.74
Smoked at least once in last week	3.30	2.56, 4.26	1.73	0.96, 3.14
Current smoker	3.44	2.57, 4.59	1.91	0.94, 3.89
Violent crime	1.55	1.03, 2.32	2.34	1.05, 5.25

[1] Coefficients for early-reproducing daughters (n=302) relative to non-reproducing daughters (n=1782)

2 Coefficients for daughters of low-capital mothers (n=72) relative to daughters of high-capital mothers (n=399)

Categorical variables assessed by chi-square test (expressed as odds ratio (OR) and 95% confidence intervals (CI)

Continuous variables assessed after natural log transformation by independent-samples t-test (multiplied by 100%, expressed as % difference) and 95% confidence intervals (CI)
